# Anticancer, antioxidant and antibacterial potential of L-Glutaminase (*Streptomyces roseolus* strain ZKB1) capped silver and zinc oxide nanoparticles and its molecular characterization

**DOI:** 10.1186/s40643-025-00857-w

**Published:** 2025-03-23

**Authors:** Zabin K. Bagewadi, Gouri H. Illanad, T. M. Yunus Khan, Shaik Mohamed Shamsudeen, Sikandar I. Mulla

**Affiliations:** 1 Department of Biotechnology, KLE Technological University, Vidyanagar, Hubballi, Karnataka 580031 India; 2 Department of Mechanical Engineering, College of Engineering, King Khalid University, 61421 Abha, Saudi Arabia; 3 Department of Diagnostic Dental Science and Oral Biology, College of Dentistry, King Khalid University, 61421 Abha, Saudi Arabia; 4 Department of Biochemistry, School of Applied Sciences, REVA University, Bangalore, 560064 India

**Keywords:** L-Glutaminase, Nanoparticles, Immobilization, Analytical characterization, Biological activities

## Abstract

The current investigation reports anti-cancer, antioxidant and antibacterial potential of L-Glutaminase (*Streptomyces roseolus* strain ZKB1) and L-Glutaminase capped nanoparticles. The highest L-Glutaminase production of 9.57 U/mL was achieved on the 4th day of fermentation when L-Glutamine was used as the sole carbon and nitrogen source. Enhanced recycling stability was observed after 6 cycles using L-Glutaminase immobilized in 3% agar and agarose matrices. Free and immobilized L- Glutaminase showed *K*_*m*_ of 13.89 ± 0.8 and 7.13 ± 0.3 mM and *V*_*max*_ of 18.40 ± 1.5 and 24.21 ± 1.7 U/mg respectively. L- Glutaminase capped silver (AgNP) and zinc oxide (ZnONP) nanoparticles were synthesized and structurally characterized using UV visible spectroscopy, FTIR, SEM–EDS, XRD and AFM. L- Glutaminase capped AgNP and ZnONP exhibited good thermal stability with five and three stages weight loss pattern respectively based on TGA. L-Glutaminase capped AgNP exhibited highest inhibitory activity against *B. subtilis* (45 $\pm$ 0.5 mm) and *E. coli* (33 $\pm$ 0.8 mm) whereas, L-Glutaminase capped ZnONP demonstrated highest inhibition against *E. coli* (30 $\pm$ 0.3 mm) and *B. cereus* (25 $\pm$ 0.5 mm). Increased nanoparticles concentration exhibited increased inhibitory potential as compared to wild L-Glutaminase and lowest MIC of 0.09 µg/mL was exhibited against *B. cereus*. L-Glutaminase capped nanoparticles demonstrated significant antioxidant properties through *in-vitro* ABTS and DPPH radical scavenging assays in a dosage-dependent manner. L-Glutaminase and capped AgNP and ZnONP, demonstrated pronounced cell cytotoxicity against MCF-7 cancerous cell line with 57.17 µg/mL, 8.13 µg/mL and 28.31 µg/mL IC_50_ values respectively, suggesting promising properties as anticancer agents in enzyme-based therapy. The results reveal promising biological activities with potential applications in healthcare sector.

**Graphical Abstract:**

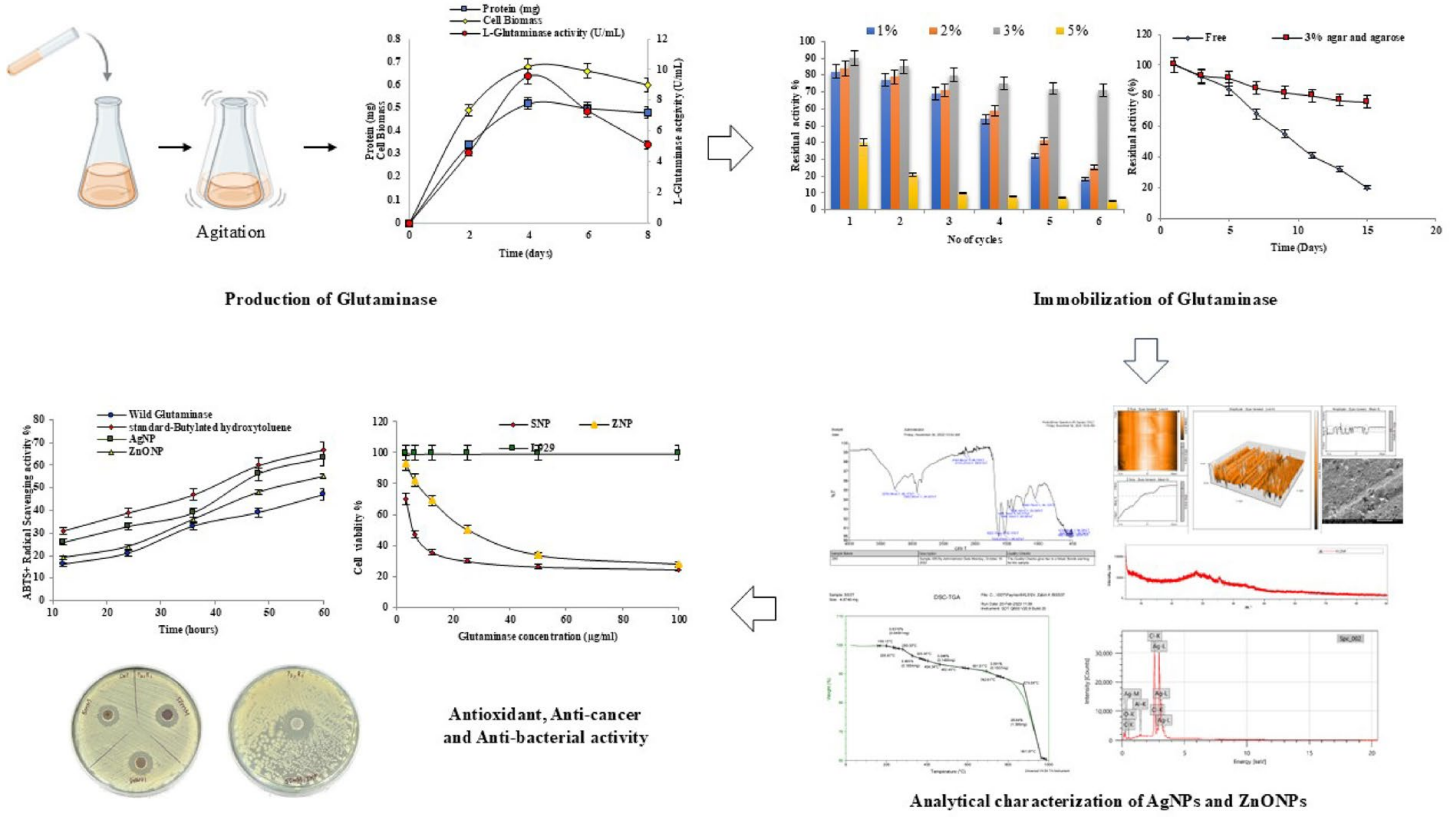

## Highlights

- L-Glutaminase (Streptomyces roseolus strain ZKB1) immobilized in agar and agarose matrix showed enhanced recycling stability.
- L-Glutaminaseand its capped AgNPs and ZnONPs demonstrated potential anticancer, antioxidant and antibacterial properties.
- L-Glutaminase capped AgNPs and ZnONPs were structurally characterized by UV-Vis, FTIR, SEM-EDS, XRD and AFM.

## Introduction

Carcinogenesis is the development of the cancer which is characterized by the transformation of normal cells into malignant tumors. According to World Health Organization nearly 10 million deaths were reported in the year 2020 owing to cancer. Cellular and molecular pathways in cancer cells are reprogrammed to maintain and support the uncontrollable proliferation (Cyriac and Lee 2024). Cancer cells are metabolically characterized by increased glutamine metabolism and large requirements of glutamine as energy source for cell proliferation and cell signaling regulation. The scarcity of glutamine can aid in starvation of cancer cells and restrict its proliferation rate (Blachier et al. 2023). L-Glutaminase (EC.3.5.1.2) is an enzyme classified within the amidohydrolase family, a phosphate activated enzyme which cleaves glutamine into glutamic acid and ammonia (Bagewadi et al. 2024). It also functions as nitrogen and carbon source for the biosynthesis of amino acids and lipids respectively (Mirshekaran et al. 2024). As, glutaminase functions to hydrolyze glutamine, it creates scarcity of glutamine to cancer cells thereby building up oxidative stress and further leading to cancer cell death. This aids in developing glutaminase as a therapeutic anticancer enzyme for treatment of cancer. Recently, enzyme based cancer treatment was revealed as a prospective strategy owing to its specific operational mechanism.

Pathogenic microbial infections have posed tremendous challenges to human health worldwide causing respiratory and intestinal diseases, fatigue, headache etc. *Staphylococcus aureus* cause high pathogenicity through the release of virulence factors and is also involved in triggering life-threatening diseases (Zang et al. 2024).Traditional antibiotics are commonly employed to cater to the infections. Antibiotics act through various mechanisms such as, in *Escherichia coli* by inhibiting replication and transcription processes through interaction with DNA gyrase and topoisomerase IV*.* However, the growing drug-resistant strains and genetic mutations have made the treatment incompetent thereby imposing demand for investigation of inventive solutions (Gao et al. 2024). A range of variety of microbes, like actinomycetes, bacteria, yeasts, and fungi serve as sources for glutaminase enzymes (Wardah et al. 2023). These microbial glutaminases differ in their physicochemical and biochemical properties.Despite the diverse biochemical properties in glutaminases produced by actinomycetes, these enzymes have majorly isolated from the *Streptomyces* genus within this group (Amobonye et al. 2019).The large-scale industrial application of enzymes is hampered by the limitations in their operating stability and the complexity associated in their post-reaction recovery and subsequent redeployment. To overcome these limitations, to improve the durability of the enzyme and to target the potential deliveries, immobilization of enzyme plays a crucial role (Kondrat et al. 2022). Enzyme immobilization methods can be broadly classified as physical and chemical. Physical immobilization methods, such as entrapment or encapsulation, contain the enzyme within a matrix while keeping it physically isolated from the reaction liquid. Chemical immobilization refers to the direct interaction between enzyme and solid matrix or support through chemical interaction that includes stable covalent bonds (Romero-Fernández and Paradisi 2020). Immobilization of enzymes transforms industrial processes by allowing enzyme reusability while lowering costs and consumption. Furthermore, it improves enzyme stability against environmental changes like pH and temperature, extending activity and facilitating continuous catalysis for increased process efficiency (Homaei et al. 2013). Immobilized enzymes ease downstream processing by allowing for easier separation and greater control over reaction conditions, resulting in improved product quality and process control. This adaptability broadens their applications across various fields (Bashir et al. 2020). Enzyme immobilization finds applications across medicines, detergents, chemicals, textiles and food processing (Maghraby et al. 2023). Immobilized enzymes are utilized in biomedical and therapeutic applications, wastewater treatment for bioremediation, and the manufacturing of active packaging materials. They provide improved stability, reusability, and efficiency in several of industrial processes (Najim et al. 2024). Multiple enzymes are confined on scaffolds to achieve higher catalytic competencies as compared to free enzymes. (Cao et al. 2019) developed a model of artificial multi-enzyme catalyst by co-encapsulating multiple enzymes in metal–organic framework nanocrystals as a promising enzyme immobilization strategy with enhanced activity of multiple enzymes owing to the smaller distance between enzyme molecules. This suggests the applications of enzyme designing in synthetic biology systems. Nanotechnology is rapidly developing new uses, including the ability to synthesize nanoparticles of different shapes and sizes (Garg et al. 2020). Metal nanoparticles have been developed from many biological systems (Karunakaran et al. 2023). Nanoparticles synthesis can be broadly classified into two primaryways. Chemical synthesis offers a diverse set of techniques for producing large number of nanoparticles including, chemical reduction (Szczyglewska et al. 2023), photochemical (Jara et al. 2021), physiochemical (Sharma et al. 2020), electrochemical (Kuntyi et al. 2020), microwave irradiation (Pauzi et al. 2019), microemulsion (Sun et al. 2019), and laser ablation (Rashid et al. 2021). While they offer advantages they rely on toxic ingredients and often require expensive and labor-intensive equipment. Biological synthesis, also called as green synthesis presents an alternative. This method relies on natural products like plant extracts, microorganisms, or enzymes. This offers a more economical and sustainable friendly alternative. The increasing preference for the biological synthesis is due to its exhibiting distinct physiochemical properties, environmental stability and potential for tailored nanoparticle properties (Iravani et al. 2014). Some constraints related to the synthesis of nano-composites using enzymes and sliver nanoparticles are, the construction of stable enzyme-nanocomposites preserving high activity and understanding the synergistic mechanism of enzyme-nanocomposites. A study by (Xiong et al. 2022) projected a rationally designed lysozyme-Ag-polymer nanocomposites that displayed enhanced stability in a cooperative manner and proposed a tightly encapsulated invasion bactericidal mechanism where the bacteria intimately interacted with nanocomposites and lysozyme and silver executed the killing effect hence, suggesting its biocompatible attribute with mammalian cells. (Li et al. 2016) reported a novel hybrid catalyst such as, enzyme–copper phosphate composite that was prepared by self-assembly-disassembly approach with high enzymatic and copper-catalytic activities for chemo-enzymatic cascade reactions. A study (Hou et al. 2017) demonstrated the synthesis of enzyme–metal–organic framework composites using ink-jet printing bio-inks such as, Cyt c–ZIF-8 composites were developed on filter paper revealing high activity for detection of hydrogen peroxide.

The current study evaluates the biological attributes of L-Glutaminase produced from *Streptomyces roseolus* strain ZKB1 and L-Glutaminase capped nanoparticles. The research focuses on L-Glutaminase production and its immobilization for enhanced stability and improved kinetics. L-Glutaminase capped nanoparticles were synthesized and characterized using analytical techniques. L-Glutaminase and its nanoparticles were examined for potential antibacterial, antioxidant and anticancer properties. The outcomes suggest the prospective applications of the study in cancer treatment.

## Materials and methods

### Chemicals and substrate

All of the chemicals (agar, agarose, silver nitrate, zinc acetate dehydrate, 2,2–Azinobis– (3–Ethylenebenzothiozoline–6–Sulfonic Acid), α, α-Diphenyl-β-picryl-hydroxyl) and substrates (L-glutamine) utilized in this present study were acquired from Merck and Co. Inc. (USA) and Sigma-Aldrich Pvt. Ltd. (USA). Microbial cultures for antibacterial studies were procured from National Collection of Industrial Microorganisms (NCIM), Pune and Microbial Type Culture Collection and Gene Bank (MTCC), Chandigarh. Human breast cancer cell line MCF-7 and normal mammalian cell line L929 were employed for anticancer activities.

### Isolation and molecular sequencing of L-Glutaminase strain

The bacterial strain producing L-Glutaminase was obtained during isolation employing the source of soil from ant hill surrounding region of Hubballi, Karnataka, India. Serial dilution method and subsequent plating was adopted to isolate pure culture of L-Glutaminase producers on minimal glutamine agar medium containing (g/L) NaCl 1.0; KH_2_PO_4_1.0;KCl 0.5;FeSO_4_ 0.1; L-glutamine 10; MgSO_4_ 0.5; ZnSO_4_ 0.1; Phenol red 0.12 (Gomaa 2022). 50 μg/mL of cycloheximide was added to avoid fungal contamination. L-glutamine supported the nitrogen and carbon requirements. Phenol red was employed as a pH indicator. The bacterial colonies were observed by incubating the plates at 35 ± 2 ºC (2–3 days). Isolated colonies were primarily screened based on the change in color of media. The isolates that demonstrated the formation of pink colored zones were concluded to be positive L-Glutaminase producers. The change of color indicated the utilization of L-glutamine and subsequent ammonia accretion. Purified colonies showing positive for L-Glutaminase producers with maximum extend of color change was chosen for further analysis. Purified potential strain ZKB1 was preserved on slant (4 ºC) and employed for 16S rRNA molecular characterization by adopting previously reported method (Bagewadi et al. 2022). Briefly, using Veriti^®^ 96-well Thermal Cycler (Applied Biosystems, USA), the genomic DNA was extracted and amplified. Amplification was executed using gene-specific primers (27F forward primer; 1492R reverse primer). Standard conditions for polymerase chain reaction (PCR) amplification were employed (Bagewadi et al. 2022) containing a PCR reaction comprising of Taq, primer and DNA template. The obtained PCR product was purified and subjected for sequencing. The alignment of sequences was carried out. The maximum likely related organism’s nucleotide sequences were obtained from database (EzBioCloud) and distance matrix was generated using Ribosomal Database Project. Neighbour-joining technique (MEGA6 software) constituting 500 bootstrap replicates was executed to construct the phylogenetic tree to locate the nearest resembling bacterial strains (Revankar et al. 2023a).

### L-Glutaminase production from isolated bacterial culture

L-Glutaminase production from isolated *Streptomyces roseolus* strain ZKB1 was conducted in 250 mL Erlenmeyer flasks. Fermentation kinetics was monitored over an 8 days period containing 100 mL of production medium. The medium was consisted of (g/L): L-glutamine 1.0; yeast extract 1.0; MgSO_4_ 0.5; D-glucose 1.0; KH_2_PO_4_ 0.5; NaCl 1.0; K_2_HPO_4_ 0.5. A loopful of a 20 h old culture was added to 10 mL of nutrient broth to prepare the inoculum. The inoculum was subsequently incubated for 16–18 h at 37 °C, with continuous agitation of 200 rpm using a Scigenic Biotech (India) incubator. A 2% inoculum was introduced into 100 mL of sterile production medium (pH 7.0) in aseptic condition, contained within a 250 mL Erlenmeyer flask. The cultivation was conducted with orbital shaking at 200 rpm at 37 °C for duration of five days. Following incubation, the culture broth was introduced to centrifugation at 10,000 rpm at 4 °C for 10 min. to separate the biomass (pellet) from the supernatant. The biomass was subsequently re-suspended in saline, and optical density measurements were determined at 660 nm. The supernatant was employed as enzyme source for the estimation of L- Glutaminase by executed L- Glutaminase assay as described below.

### L-Glutaminase assay and analysis of protein

To measure ammonia production, a reaction mixture (test samples) was prepared by combining 0.5 mL of 0.04 M L-glutamine in phosphate buffer of pH 7.0, 0.5 mL of 0.5 M phosphate buffer, and 1.0 mL of enzyme supernatant (collected on days 1–5 and stored at 4 °C). The final reaction volume was set to 2.0 mL. To facilitate enzyme-catalyzed ammonia release, the mixture was incubated 30 min. at 37 °C. To terminate the reaction and precipitate proteins, 0.5 mL of 1.5 M trichloroacetic acid was added to the reaction mixture. The mixture was then centrifuged at 10,000 rpm for 10 min. to separate the precipitated proteins. The supernatant containing the released ammonia was reacted with Nessler’s reagent of 0.5 mL and incubated at 20 °C for 20 min. to form a colorimetric complex. Finally, the absorbance of the supernatant at 450 nm was measured to determine ammonia concentration (Bagewadi et al. 2024). The blank constituted of the same reaction mixture (2.0 mL), however the enzyme supernatant employed in the blank was heat denatured (boiling water bath for 30 min.) before adding to the reaction mixture followed by incubation under the similar conditions as test samples and addition of Nessler’s reagent. Blank was employed to set the spectrophotometer to zero (background correction) at 450 nm to capture the absorbance of the test samples. The liberation of ammonia was calculated from the standard graph using ammonium sulfate standard. One unit of L-Glutaminase activity was defined as the amount of enzyme required to release 1 µmol of ammonia per minute per mL (µmole/mL/min.) under defined assay conditions and expressed as U/mL.

The soluble total protein content (mg) of L-Glutaminase (enzyme) was estimated according to standard method of Lowry et al. (1951) using protein standard as BSA. All the analysis were executed in triplicates and presented at mean ± standard deviation.

### Immobilization of L-Glutaminase and stability assessment

Immobilizing the enzyme is critical for increasing its stability and targeting possible delivery. A method described by (Prakash and Jaiswal 2011) was employed for immobilization of L-Glutaminase. Agar and agarose (1, 2, 3 and 5%) combined matrix was prepared in the ratio of 1:1 in a phosphate buffer (0.1 M), pH 7.0 at 50 ºC. After cooling, L-Glutaminase of 1.0 mL was mixed with 9.0 mL of respective agarose and agar concentrations separately and immediately casted onto glass petri plates. The solution was allowed to solidify and subsequently cut down into small pieces of 5 mm cubes and washed thoroughly to remove any unbound enzyme. Finally, the cubes were preserved in 0.1 M phosphate buffer at a pH 7.0 and stored at 4ºC. The efficiency of immobilization (EF) was estimated using following equation (Bagewadi et al. 2017).

$${\text{EF}}\,\left( \% \right) \, = \, \left( {{{Ai \, } \mathord{\left/ {\vphantom {{Ai \, } {Af}}} \right. \kern-0pt} {Af}}} \right) \times {1}00$$

where *A*_*i*_ represents the specific activity of an immobilized enzyme computed by subtracting the specific activity of the unbound enzyme from the specific activity of the enzyme (*A*_*f*_).

The efficacy of immobilized agar and agarose L-Glutaminase cubes were determined by assay procedure as described above. Briefly, 2–3 cubes were incubated with L-glutamine solution at room temperature for 30 min. and allowed for substrate interaction with immobilized enzyme. The absorbance measured at 450 nm was directly proportional to the concentration of ammonia in the sample (Tork et al. 2018). The immobilized L-Glutaminase (1, 2, 3 and 5%) was recycled and its stability was determined after each cycle for 6 consecutive cycles. Immobilized L-Glutaminase was obtained after the reaction and washed with assay buffer to reuse for further cycles. The residual activity of immobilized L-Glutaminase (1, 2, 3 and 5%) was calculated after each cycle. The free enzyme was considered as 100%.The activity of the immobilized enzyme during each cycle (reuse) was calculated as residual activity and compared with free enzyme using following equation.

$${\text{Residual activity }}\left( \% \right) \, = \, \left( {{\text{Obtained activity }}/{\text{ Initial activity}}} \right) \, \times { 1}00$$

Initial activity represents the activity of free enzyme and obtained activity represents the activity obtained after each cycle of reuse.

The storage stability of immobilized L-Glutaminase (agar and agarose 3%) was also evaluated for a period of 15 days by monitoring L-Glutaminase activity. The stability of free and immobilized enzymes is shown as residual activity %. The residual activity on first day was considered as 100%.

### Kinetics of free and immobilized L-Glutaminase

The Lineweaver–Burk diagram was utilized to compute the kinetic parameters of free and immobilized (agar and agarose 3%) L-Glutaminase, including maximum velocity (*V*_*max*_), Michaelis–Menten constant (*K*_*m*_), turnover number (*k*_*cat*_), and catalytic efficiency (*k*_*cat*_*/K*). The equations are as suggested by (Kerouaz et al. 2021). *k*_*cat*_ was calculated using the molecular weight of enzyme ~ 52 kDa (Data not shown). The enzyme's catalytic activity was assessed at concentrations ranging from 25 to 100 mM, following standard experimental protocols.

The Turnover number *K*_*cat*_ was determined using the following equation:

$${\text{K}}_{{{\text{cat}}}} \, = \,\frac{Vmax}{{\left[ E \right]}}$$

In this equation, *V*_*max*_ denotes the maximum reaction velocity attainable under saturating substrate conditions, while *[E]* represents the concentration of active enzyme.

### Synthesis of L-Glutaminase capped silver (AgNP) and zinc oxide (ZnONP) nanoparticles

The biogenic synthesis of potential nanoparticles can be accomplished by employing intracellular or extracellular biological molecules derived from microbes. AgNP have unique properties like high surface area that exhibits antimicrobial activity for fighting infections. L- Glutaminase capped AgNP were synthesized by previously reported method (Revankar et al. 2023b). Silver nitrate solutions of 5 mM, 10 mM and 50 mM concentrations were prepared by continuous stirring for 30 min. on a magnetic stirrer. Subsequently, L-Glutaminase of 0.5 mL was combined with 4.5 mL of silver nitrate solution of defined concentrations. All mixtures were kept under UV light for 10 min. to initiate the formation of nanoparticles. Following the UV exposure, the reactions were microwaved for 30 s. to induce the color change to brownish, confirming the synthesis of AgNP. The brownish precipitate was separated from the supernatant and was rinsed twice with deionized water to eliminate any ions. UV–Vis spectroscopy was utilised to analyse the synthesized AgNP absorbance properties. The synthesized L-Glutaminase capped AgNPs were subjected to centrifugation for 20 min. at 10,000 rpm to concentrate them. The resulting pellet was collected and dried for further analytical characterization.

Synthesis of L-Glutaminase capped ZnONP were executed that offer excellent UV protection and have high potential use in electronics and catalysis due to their unique electrical and light-responsive nature. The L-Glutaminase capped ZnONP of concentration 10 mM and 50 mM were synthesized by previously reported method (Alahdal et al. 2022). The mixture of L-Glutaminase (0.3 mL) and zinc acetate dehydrate (0.7 mL) was incubated for 15 min. at 60 ºC to initiate the reaction and synthesize ZnONP, and then incubated for next 24 h at room temperature. The aqueous Zn^2+^ was reduced to ZnONP which was triggered by the addition of protein (L-Glutaminase) derived from *Streptomyces roseolus* strain ZKB1. This reducing effect of protein present in the supernatant is responsible for biogenic synthesis of ZnONP. The visual examination for formation of white precipitate indicates the synthesis of ZnONP The absorbance of the L-Glutaminase capped ZnONP was studied by employing UV–Vis spectroscopy. The synthesized nanoparticles were subjected to centrifugation for 20 min. at 10,000 rpm to concentrate them. The resulting pellet was collected and dried for further characterization. Additionally, the L-Glutaminase residual activity of L- Glutaminase capped AgNP and ZnONP (50 mM) was verified according to the assay method described above.

### Analytical characterization of synthesized nanoparticles

#### Fourier transform infrared spectroscopy (FTIR) analysis

Fourier transform infrared spectroscopy acts as a versatile approach in identifying the chemical functional groups and analyse both the organic and inorganic compounds by understanding the material’s ability to absorb the infrared light. The functional groups of L-Glutaminase capped AgNP and ZnONP was investigated using FTIR (Perkin Elmer, FTIR1760) by employing previously reported method (Bagewadi et al. 2016a). The dried nanoparticles were powdered and mixed with potassium bromide to prepare KBr pellets, which were then scanned in the spectral range of 4000–450 cm^−1^ range at a resolution of 1.0 cm^−1^.

#### Thermogravimetric analysis (TGA) analysis

TGA (Perkin -Elmer USA) was adopted to measure the thermostability of the L-Glutaminase capped AgNP and ZnONP by measuring their change in mass under controlled heating conditions. A 5 mL sample of powdered nanoparticles were allowed for heating under controlled conditions of nitrogen at temperatures ranging from 10-1000ºC at a rate of 10ºC min^−1^ (Bagewadi et al. 2020).

#### Scanning electron microscope (SEM) with energy-dispersive X-ray (EDX) analysis

To investigate uniformity and surface features of synthesized L-Glutaminase capped AgNP and ZnONP, SEM–EDS (VEGA\TES-CAN, USA) was employed according to the report published earlier (Bagewadi et al. 2016b). Nanoparticle samples were placed on aluminum stubs under vacuum followed by gold coating. The elemental composition of the nanoparticles was analysed using X-ray microanalysis system (EDX). This technique excites atoms within the nanoparticles, causing them to emit distinctive X-rays that can be utilised to determine the elemental composition of the material.

#### X-ray diffraction (XRD) analysis

The crystal structure and size of synthesized L-Glutaminase-capped AgNPs and ZnONPs were analyzed using an X'Pert PRO P analytical diffractometer. X-Ray diffraction patterns were collected over a 2θrange of 5º to 90º with a scanning frequency of 10.00º/min.ata current of 30 mA and voltage of 40 kV (Revankar et al. 2023b).

#### Atomic force microscopy (AFM) analysis

Atomic force microscopy, specifically a Bruker MM8 system, was used to characterize the surface morphology and size of the synthesized nanoparticles. To prepare samples for imaging, the L-Glutaminase capped AgNPs and ZnONPs were sediment to centrifugation at 5000 rpm for 10 min. The resulting pellets were washed thoroughly with deionized water and re-suspended. Subsequently, drops of the re-suspended particles were deposited on glass side, spread into thin smear and air-dried. Finally, the prepared smears were analyzed by AFM to capture high-resolution images (Saravanan et al. 2017).

### Functional characterization of L-Glutaminase

#### Antibacterial activity of L-Glutaminase and synthesized L-Glutaminase capped nanoparticles

Antibacterial activity of an enzyme and its nanoparticles acts as a shield against infections, ranging from common illnesses to severe ones. The antibacterial activity of L-Glutaminase, AgNP at concentration of 5 mM, 10 mM and 50 mM and ZnONP at concentration of 50 mM was evaluated using well diffusion method following a previously established protocol (Revankar et al. 2023b). This study investigated antibacterial property by testing against six pathogens, including Gram-positive bacteria *Bacillus subtilis, Staphylococcus aureus* and *Bacillus cereus* and the Gram-negative bacteria *Pseudomonas aeruginosa, Escherichia coli* and *Salmonella typhimurium*. The selected pathogens were previously cultured in nutrient broth medium and incubated for 24 h. at 37 ºC. The freshly grown pathogenic cultures (24 h) were individually streaked on nutrient agar plates and wells were created to load 100 µL aliquots of L-Glutaminase, AgNP and ZnONP respectively. The sample loaded culture plates were incubated for 24 h at 37 ºC to obtain the zone of inhibition against the tested pathogens. Following incubation, the diameter of any clear zones surrounding the wells (zones of inhibition) was measured (mm). Standard cefixime was considered for the positive control.

Clinical and Laboratory Standards Institute (CLSI) guidelines were incorporated to study the minimum inhibitory concentration (MIC) of wild L-Glutaminase and L-Glutaminase capped AgNP and ZnONP using micro broth dilution method. The six different bacterial cultures listed above, representing both Gram-positive and Gram-negative, were separately cultured in Mueller–Hinton broth and allowed to grow at 37 ºC overnight at 150 rpm to achieve the exponential growth. MIC was carried out as reported previously (Shettar et al. 2023b). The cultures were then adjusted to achieve a standardized concentration of 10^7^ CFU/mL using fresh broth. Subsequently, various L-Glutaminase concentration (90, 45, 18, 9, 4.5, 1.8, 0.9, 0.6, 0.45, 0.22, 0.09 and 0.06 μg/mL) and AgNP and ZnONP concentration (50, 25, 12.5, 6.25, 3.125 and 1.56 mM) were prepared in sterile deionized water and then added to the bacterial culture. The broths were then inoculated for 24 h. at 37ºC and spectrophotometer was utilized to assess bacterial growth by measuring the absorbance at 600 nm. For positive control and negative control, uninoculated broth without desired samples and inoculated broth without desired samples was considered respectively. The MIC of both wild L-Glutaminase (µg/mL) and the capped nanoparticles (mM) was identified as the lowest concentration that inhibited visible bacterial growth after 24 h. incubation period.

#### Antioxidant activity of L-Glutaminase and synthesized L-Glutaminase capped nanoparticles

##### 2,2–Azinobis–(3–ethylenebenzothiozoline–6–sulfonic acid) (ABTS) assay

The antioxidant property of purified L-Glutaminase and synthesized L-Glutaminase capped nanoparticles were tested for the radical cation by ABTS scavenging method using the method previously reported (Bagewadi et al. 2019). An ABTS radial cation solution was prepared by incubating a reaction mixture of ABTS and potassium persulfate in the dark conditions for 16 h. The solution was then diluted and its absorbance adjusted to 0.75 at 734 nm to ensure consistency for the assay. 100 µl of purified L-Glutaminase, AgNP (50 mM) and ZnONP (50 mM) were incubated separately with 1.0 mL of ABTS solution for 30 min. at constant temperature of 25 ºC. The level of free radical scavenging was assessed based on the reduction in absorbance at 734 nm. The standard used was butylated hydroxytoluene (BHT). The scavenging activities were recorded for 12–60 h time period.

##### α, α-Diphenyl-β-picryl-hydroxyl (DPPH) radical scavenging assay

The DPPH radical scavenging assay, as outline by (Bagewadi et al. 2019) was utilized to validate the antioxidant properties of the samples. A 0.135 mM (1.0 mL) of DPPH solution prepared in ethanol and 1.0 mL (10–50 µg/mL) of L-Glutaminase, ZnONP (50 mM) and AgNP (50 mM) was allowed to react separately at control temperature of 25 ºC for 30 min. under dark conditions. The reduction in absorbance at 517 nm was directly correlated with the sample’s free radical scavenging activity. A well-known antioxidant, Ascorbic acid (1 mM) was utilized as a positive control. The percentage inhibition of DPPH radical scavenging activity was calculated using a previously reported equation, allowing for a quantitative assessment of the antioxidant capacity of the sample (Awad et al. 2019a).

#### Evaluation of Anticancer activity by (3-[4,5-dimethylthiazol-2-yl]-2,5 diphenyltetrazolium bromide) MTT assay

The anticancer potential of L-Glutaminase, L-Glutaminase capped AgNPs and ZnONPs was assessed against human breast cancer cell line MCF-7 and normal mammalian cell line L929 using the MTT assay, following a previously established protocol (Shettar et al. 2023b). For the cytotoxicity assay, 5,000 MCF-7 cancer cells were seeded into individual wells of a 96-well microplate. These cells were subsequently given different amounts of L-Glutaminase and nanoparticles for 24 h. Following the incubation period, the cells were exposed to an array of the concentrations of 6.25 µg/mL, 12.5 µg/mL, 25 µg/mL, 50 µg/mL, 100 µg/mL and 200 µg/mL for next 48 h. Upon completion of treatment, the cells were thoroughly washed to remove residual test compounds with phosphate buffer saline (PBS). After the treatment period, each well received 0.5 mg/mL of MTT solution and was further incubated for 4 h. The resulted formazan crystals were then solubilized in dimethyl sulfoxidewhich minimizes the interference of residual MTT agent and allows clear detection at 570 nm. Untreated cells and NPs alone served as controls, representing 100% cell viability. L-Glutaminase and L-Glutaminase capped NPs cell treatment was evaluated by calculating cell viability. The obtained absorbance values of treatments were compared with the control. All treatments were performed in triplicate for better data reliability. Doxorubicin, a known chemotherapeutic drug, was used as a positive control. Determination of IC_50_ value was in accordance with (Mostafa et al. 2021).

### Statistical analysis

All the experiments were conducted in triplicate, and the data was analyzed using Microsoft Excel. All results are presented as mean ± standard deviation (SD) and error bars indicate the standard deviation.

## Results and discussion

### Isolation and identification of L-Glutaminase strain

**Fig. 1:**
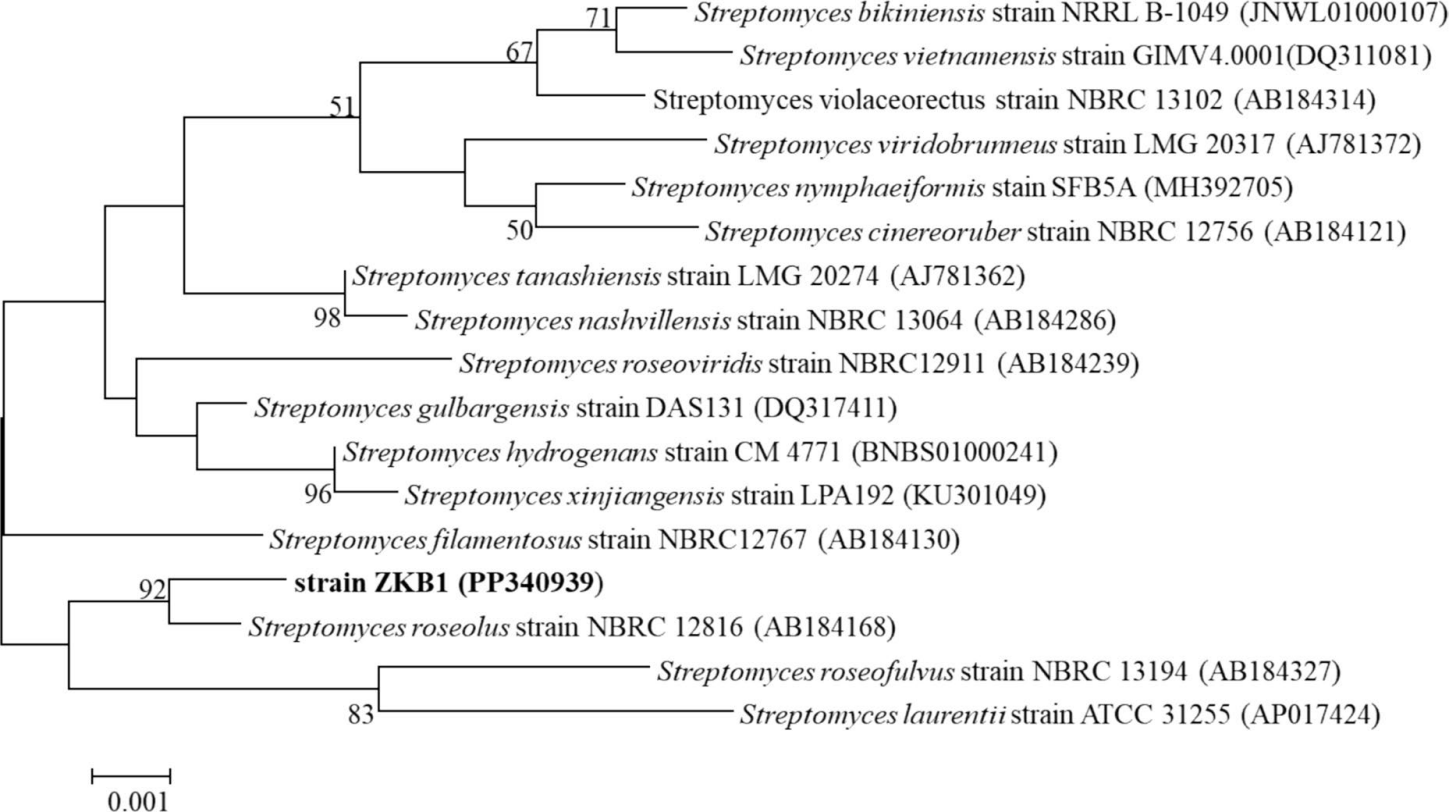
Phylogenetic tree edifying the location of strain ZKB1 (*Streptomyces roseolus* strain ZKB1 with accession number PP340939) on the basis of 16S rRNA sequences of related organisms from NCBI with accession number provided in braces

Soil and marine sources are reported to be the principal sources harboring variety of microorganisms with biological functions. Research for new and novel microbes from the environment has been of prime importance. Commonly several bacterial species are reported for L-Glutaminase production from soil and aquatic environment (Tork et al. 2018). In the current study, L-Glutaminase producing strain was isolated from soil of ant hill location. The strain was screened qualitatively for L-Glutaminase production using minimal glutamine agar medium plate assay. The strain that showed positive color (pink) change indicated its capability to produce L-Glutaminase and was used for further characterization. The purified ZKB1 strain was characterized for identification using 16S rRNA gene sequencing method. The phylogenetic tree was built to establish the maximum likeliness with the available gene sequences from GeneBank. Nearest sequences (> 99%) were selected to construct the phylogenetic relationship. Figure 1 reveals the phylogenetic relationship among the isolated ZKB1strain with those of the similar related sequences. On the basis of phylogenetic interpretation, the strain ZKB1 was further characterized to belong to *Streptomyces roseolus* species*.* The identified ZKB1 strain was nominated as *Streptomyces roseolus* strain ZKB1. The ZKB1 strain sequence with size of 1255 bp has been submitted to NCBI database with accession number PP340939. Other researchers have isolated L-Glutaminase strains from varied sources. (Gomaa 2022) isolated halophilic *Bacillus* sp. DV2-37 from marine source. *Achromobacter xylosoxidans* strain RSHG1 was isolated from an expired L-glutamine bottle (Saleem and Ahmed 2021). *Pseudomonas nitroreducens* SP.001 an intracellular glutaminase producer was isolated from oil field (Shuai et al. 2019). *Streptomyces pratensis* NRC10 was isolated from a marine dead shrimp (Tork et al. 2018). *Streptomyces* sp. GLD25 possessing antimicrobial properties were isolated from sediments samples (Djebbah et al. 2022). Reports on production of L-Glutaminase from *Streptomyces roseolus* strain are not much available in the literature.

### Production kinetics of L-Glutaminase

**Fig. 2:**
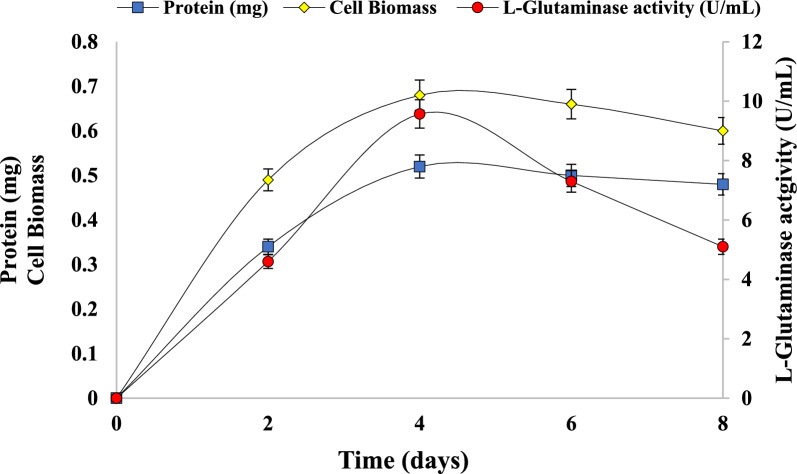
Production kinetics of the L-Glutaminase

L-Glutaminase is involved in the production of glutamic acid that is known for its flavor and nutritional property from L-glutamine substrate. The kinetics of L-Glutaminase production was studied over a period of 8 days as shown in Fig. 2. The kinetics of production revealed that the production of extracellular L-Glutaminase commenced on the 2nd day, with an initial activity of 4.6 U/mL. Subsequently L-Glutaminase activity gradually increased, reaching a peak of 9.57 U/mL on the 4th day of fermentation revealed shorter fermentation period. After the peak activity on 4th day, a decline in L-Glutaminase production was evidenced. The total protein concentration peaked at 0.52 mg on 4th day with the maximum cell biomass of 0.68 (OD). It is evident that enzyme production is dependent on the microbial growth. The level of biomass concentration directly influences the production kinetics of enzymes and also impacts the cellular metabolic functions. Significant increase in protein and cell biomass was not evidenced after 4th day. Literature suggests maximum productivity of enzymes occurs at specific fermentation period that influences growth of culture and its decline. The reduction in cell growth is linked to the nutrient’s exhaustion that exerts stress on cells and also inactivates secretary machineries requirement for enzyme synthesis (Gomaa 2022). The decline in the productivity at an extended fermentation period is possibly due to the end product of fermentation such as glutamate. Based on this kinetics, the 4th day was determined as the optimal time point for L-Glutaminase production. Our results are consistent with the reported production of L-Glutaminase from various microbial sources. Variability in L-Glutaminase fermentation period exists among most microbial strains with production starting at early stationary phase of microbial growth. The maximum activity of 5.73 U/mL was reported from wild type of *Bacillus* sp. at 32 h (Potla Durthi et al. 2019) and 5.16 U/mL in *Bacillus* sp. DV2-37 (Gomaa 2022). From *Streptomyces pratensis* NRC10, the maximum L-Glutaminase production was observed after 4 days of incubation (Tork et al. 2018)and from *Bacillus subtilis* NRRL 1315 after 48 h of incubation with 1.07 mg/mL of protein content (El-Sousy et al. 2021). Short fermentation time of 36 h was reported in *Halomonas meridian* in the later part of logarithmic cell growth phase (Mostafa et al. 2021).

### Operational stability of immobilized L-Glutaminase

**Fig. 3:**
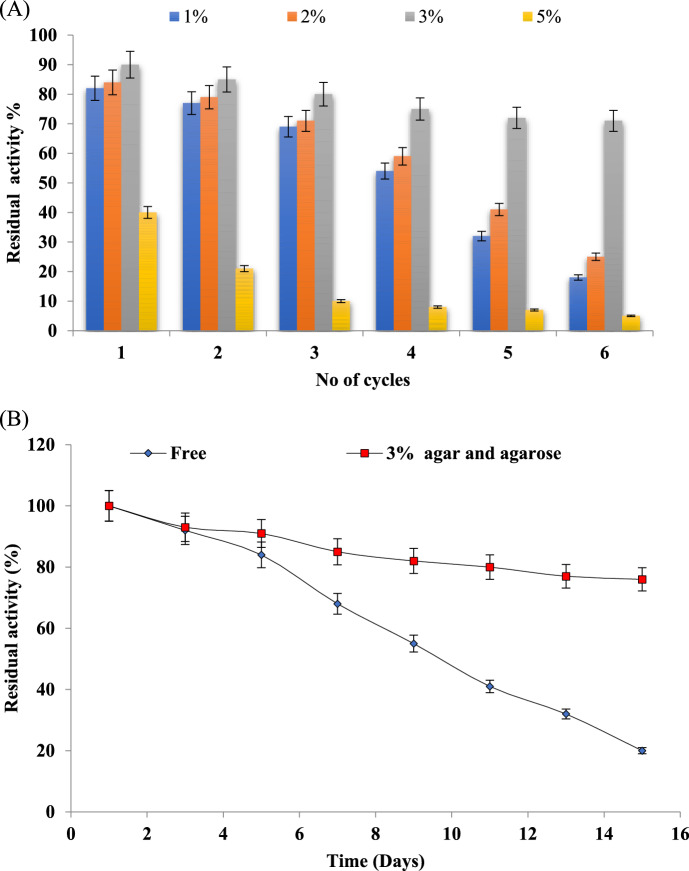
Immobilization of L-Glutaminase **A** recycling stability and **B** Storage stability

Optimal immobilization of L-Glutaminase was achieved using 3% agar and agarose matrices, resulting in the formation of stable cubes with better immobilization efficiency of 95% in comparison to other tested percentages. At 5% agar and agarose matrices, cube formation was hard. The challenges in using agar or agarose matrices at high concentrations are reported in literature (Sattar et al. 2018). At higher concentrations the matrix viscosity increases leading to hardening and improper enzyme immobilization process. The rapid gelation also hinders the in the uniform mixing process and leads to dense gel formation with smaller pores further causing diffusion problems. At higher concentration the increased mechanical strength of the matrix makes it harder and introduces irregularities. Reusability of immobilized L-Glutaminase was studied for 6 consecutive cycles for economic viability. An operational stability of 18%, 25%, 71% and 5% was recorded for 1%, 2%, 3% and 5% agar and agarose matrices respectively after 6 reusability cycles as depicted in Fig. 3A.Over the number of 6 cycles the immobilized L-Glutaminase of all tested concentrations demonstrated a decline in operational stability. However, at 3% immobilization > 70% residual activity was achieved at 6th cycle indicating its significant operational stability. The overall reduction in the activity after reuse of immobilized L-Glutaminase for consecutive cycles could be attributed to the poor binding strength of matrix and enzyme thereby leading to decline in the catalytic efficiency. Enzyme inactivation is commonly observed feature during enzyme recycling. The storage stability at 4 °C of free and immobilized L-Glutaminase was evaluated for a period of 15 days and the results are shown in Fig. 3B. Free L-Glutaminase showed an 80% loss in activity whereas; immobilized L-Glutaminase (agar and agarose 3%) revealed a 24% loss. The storage stability of immobilized L-Glutaminase(agar and agarose 3%)was significantly better with retaining > 75% of activity even after 15 days. The 3% immobilized L-Glutaminase represented the combination of agar and agarose in a composite form maintaining a structural conformation that could retain maximum enzyme, thereby providing enhanced storage stability over the tested period. (Qiao et al. 2019) demonstrated the immobilization of L-Glutaminase through covalent bonding on bamboo sticks through a glutaraldehyde modification method (L-glutaminase@BS) that enhanced the enzymatic hydrolysis with higher efficiency and extended stability. L-glutaminase@BS showed 60% activity after 5 cycles and retained 80% activity after storing for two weeks. The halo-tolerant glutaminase was immobilized onto nano magnetic cellulose sheet through protein engineering strategy that showed enzymatic hydrolysis of glutamine with 80% activity up to 5th cycle (Baskaran et al. 2018). (Kim et al. 2018) reported the immobilization of laccase with anti-proliferative properties on super-magnetized and chitosan nanotubes (Fe_3_O_4_-HNTs-CS-Lac) for possible development of anti-cancer drug.

### Evaluation of free and immobilized L-Glutaminase kinetic parameters

The enzyme kinetics of immobilized and free L-Glutaminase was studied and kinetic parameters were determined as shown in Table 1.The activity of L-Glutaminase enhanced as the concentration of the substrate (L-Glutamine) was increased till a saturation point was reached. The *K*_*m*_ value of 13.89 ± 0.8 and 7.13 ± 0.3 mM was observed for free and immobilized L-Glutaminase respectively. Lower value of *K*_*m*_ parameter indicated better specificity towards substrate binding. *V*_*max*_ of 18.40 ± 1.5 and 24.21 ± 1.7 U/mg was obtained for free and immobilized L-Glutaminase respectively. The maximum velocity of an enzyme catalyzed reaction is illustrated by *V*_*max*_. *k*_*cat*_ and *k*_*cat*_*/K*_*m*_ for free and immobilized L-Glutaminase was determined to be 35.38 min^−1^and 2.54 min^−1^ mM^−1^ and 46.55 min^−1^and 6.52 min^−1^ mM^−1^ respectively. Better catalytic efficiency was demonstrated with immobilization process. Different studies have reported variability in kinetics of free and immobilized enzymes. Similar to our findings the *K*_*m*_ was found to be 11.2 mM and 7.8 mM and *V*_*max*_ was determined to be 3.8 mM/min and 7.1 mM/min for free L-glutaminase and immobilized (L-glutaminase@BS) respectively (Qiao et al. 2019). L-Glutaminase from *Streptomyces rochei*SAH2_CWMSG and *Bacillus subtilis* OHEM11 reported a *K*_*m*_ and *V*_*max*_ of 1.314 mmol/L and 95.24 μM/min (Awad et al. 2019b) and 0.01323 mM and 94.59 UmL^−1^ min^−1^ respectively.

**Table 1:** Kinetics of L-Glutaminase from *Streptomyces roseolus* strain ZKB1

L-Glutaminase	*K*_*m*_ (mM)^a^	*V*_*max*_ (U/mg)^a^	*k*_*cat*_ (min^−1^)	*k*_*cat*_*/ K*_*m*_ (min^−1^ mM^−1^)
Free	13.89 ± 0.8	18.40 ± 1.5	35.38	2.54
Immobilized	7.13 ± 0.3	24.21 ± 1.7	46.55	6.52

^a^Data represented as mean ± standard deviation of triplicate enzymatic reactions

### UV–Visible spectroscopy characterization of nanoparticles

**Fig. 4:**
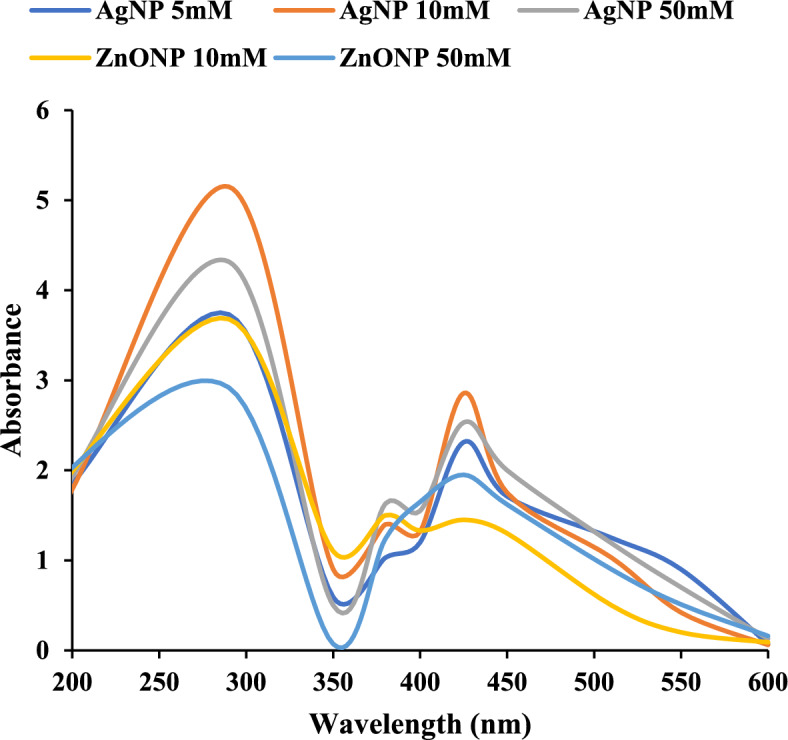
UV–Visible spectroscopy of recombinant L-Glutaminase synthesized nanoparticles

The transformation from yellow to brownish color served as a visual indicator for the successful synthesis of AgNPs. This color shift is attributed to the surface plasmon resonance (SPR) phenomenon. SPR arises from the interaction of light with the free electrons which serves as characteristic of AgNP. For the qualitative observation, UV–Vis spectroscopy was employed. The analysis confirmed the presence of AgNP with prominent peaks at 290 nm and 425 nm as depicted in Fig. 4. A color change from colorless to milky white precipitate indicated the formation of ZnONPs. UV–Vis spectroscopy confirmed the formation of L-Glutaminase capped ZnONP by revealing the absorbance peak at 280 nm. Similar study (Pallavi et al. 2022) showed a prominent peak at 418 nm for synthesized AgNPs from the culture supernatant of *Streptomyces hirsutus*SNGPA-8. Another study (Mechouche et al. 2022) silver nanoparticles synthesized from *Streptomyces tuirus* resulted in specific peak at wavelength 400 nm. Parallelly, previous research by (Sanjivkumar et al. 2019) showed prominent peak at 450 nm for silver nanoparticles synthesized by *Streptomyces olivaceus* (MSU3). In case of ZnONP, research by (Sivakumar et al. 2024) indicated the presence of zinc oxide nanoparticles from *Streptomyces sp.* at broad absorbance peak at 290 nm. Another study by (Saravanan et al. 2018) revealed zinc oxide nanoparticles formation from *Bacillus megaterium* showed prominent peak at 346 nm. (Sanjivkumar et al. 2022) investigation of zinc oxide nanoparticles synthesized from *Streptomyces spp.* exhibited highest peak at 363 nm. Additionally, the residual activity of L-Glutaminase in L- Glutaminase capped AgNP and ZnONP was verified and found to be 87% and 75% respectively. A study (Naapuri et al. 2022) reported the synthesis of nanobiohybrids demonstrating enzymatic and metallic activities in cascade reactions thereby, presenting avenues for designing novel reaction cascades.

### Analytical characterization

#### FTIR analysis of L-Glutaminase-capped nanoparticles

**Fig. 5:**
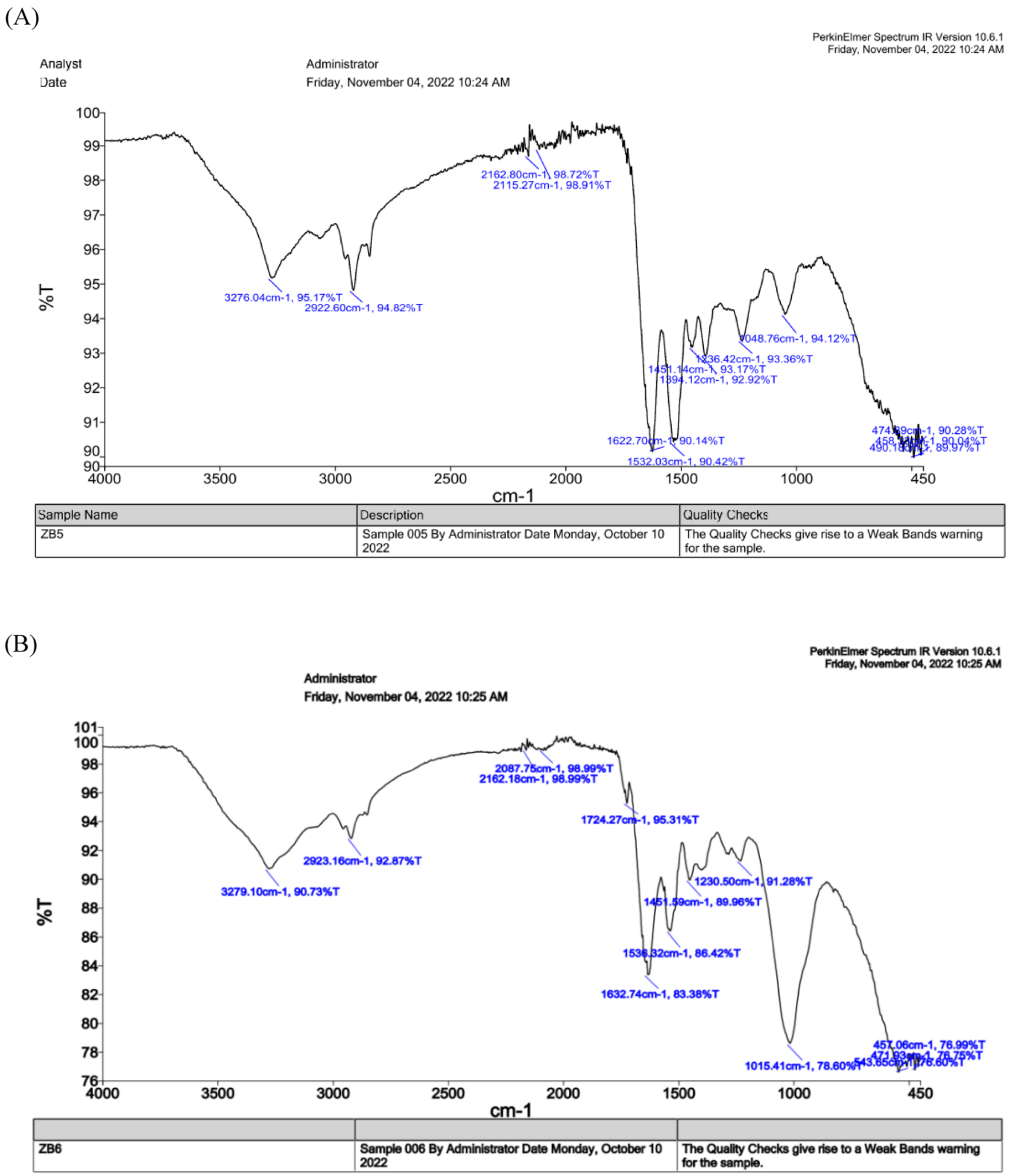

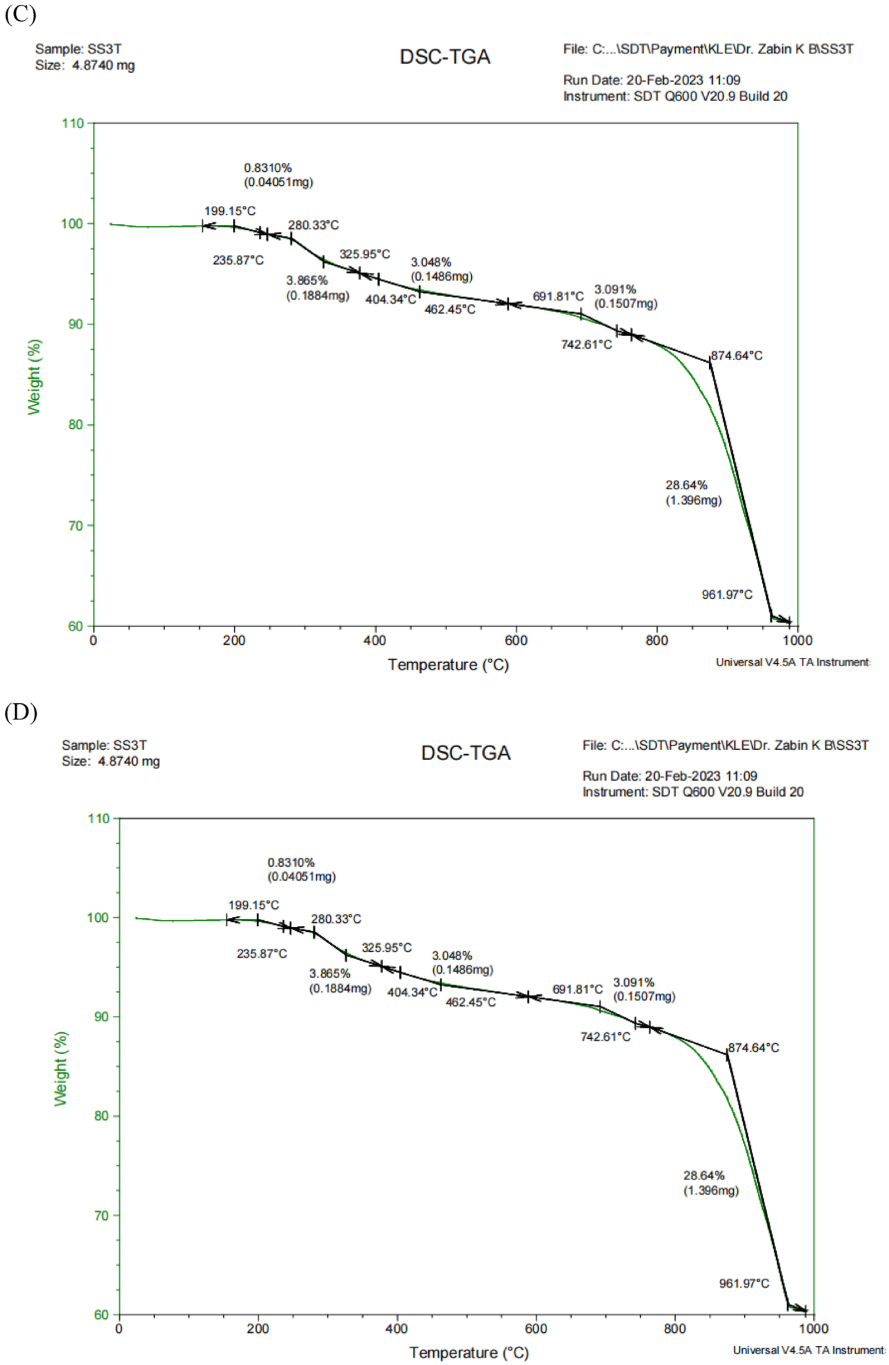

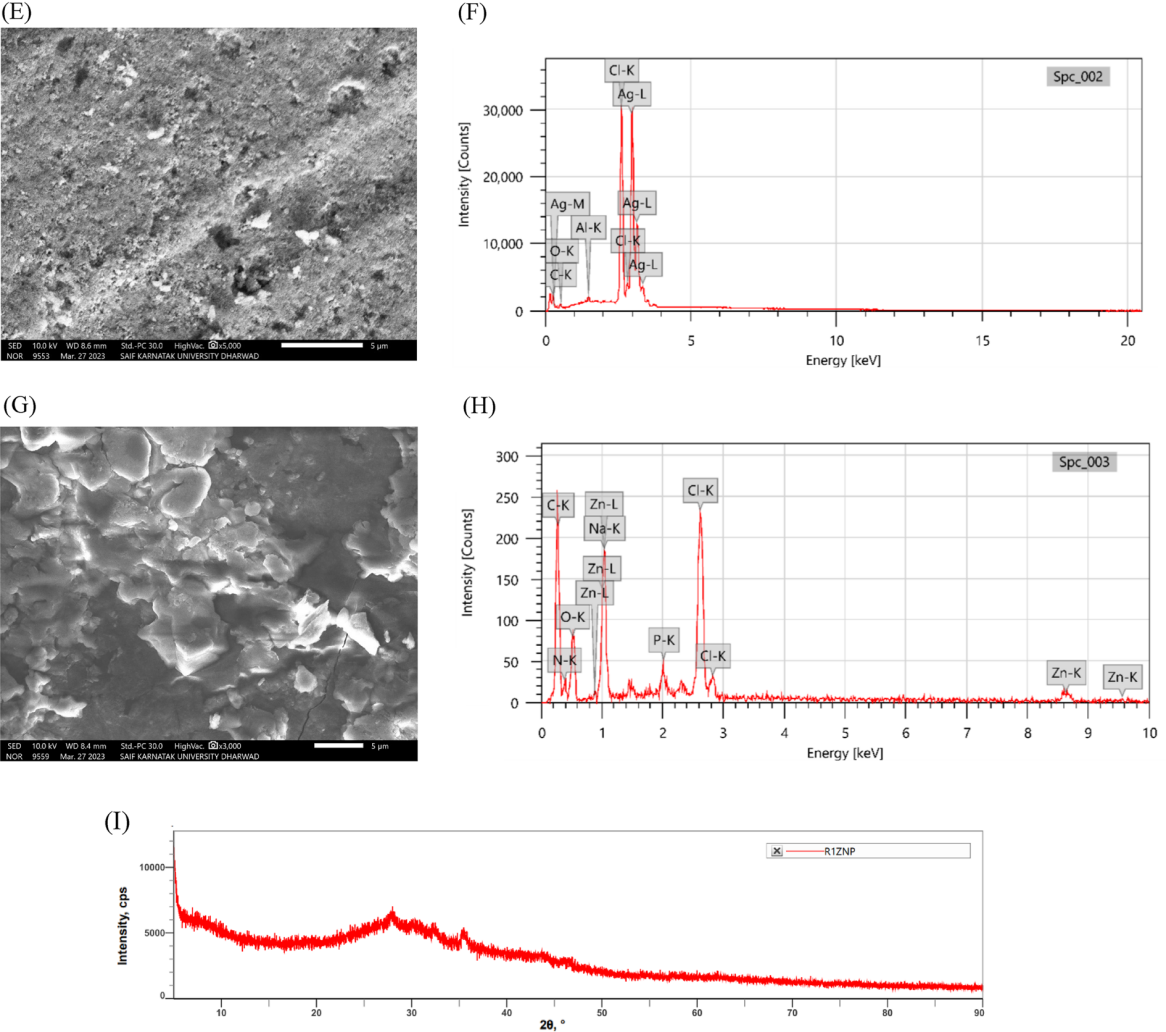

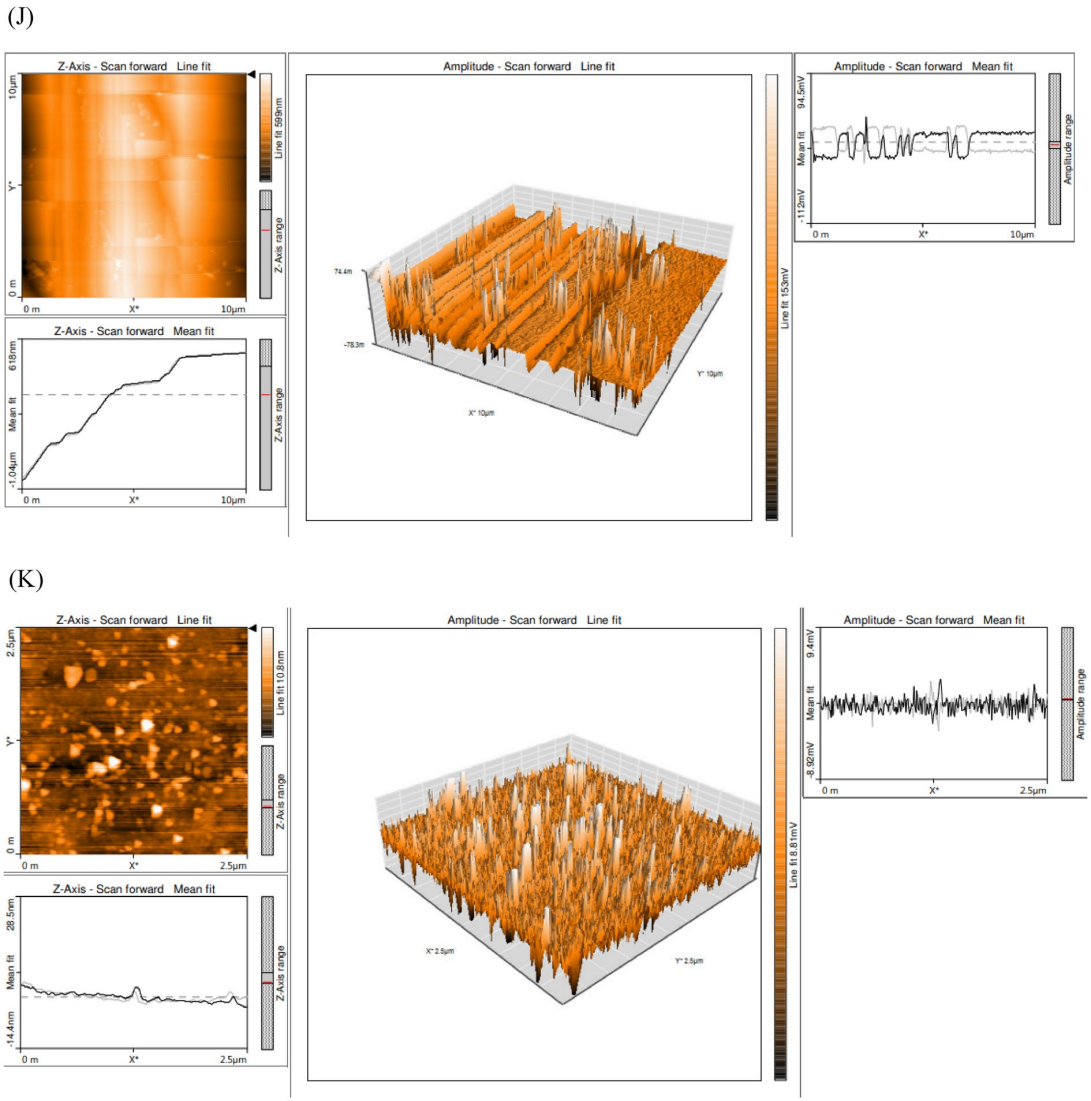
Bioanalytical characterization of purified recombinant L-Glutaminase capped nanoparticles. **A** FTIR assessment of AgNP, **B** FTIR assessment of ZnONP, **C** TGA of AgNP **D** TGA of ZnONP **E** SEM examination of AgNP, **F** EDX examination of AgNP **G** SEM examination of ZnONP, **H** EDS examination of ZnONP **I** XRD examination of ZnONP **J** AFM examination of AgNP **K** AFM examination of ZnONP

FTIR unveils the functional group on the nanoparticles and identify the interaction between the L-Glutaminase and nanoparticles responsible for prolonged stability. In this study, the IR spectra of synthesized AgNP (Fig. 5A) exhibited prominent peak at 3276.04 cm^−1^ indicating hydroxyl O–H stretches (Almalki and Khalifa 2020). Peaks at 2922.60 cm^−1^ ascertained aldehydic C-H stretching (Karthik et al. 2014). Peaks at 2162.80 cm^−1^, 2115.27^–1^ cm and 1622.71 cm^−1^ represents C $\equiv$ C alkynes and C = O ketones (Ali et al. 2023). Numerous peaks at 1532.03 cm^−1^, 1451.14 cm^−1^ and 1394.12 cm^−1^revealed C = O stretches (Revankar et al. 2023b). Peaks at 1236.42 cm^−1^ represented -O stretches (ethers) (Ali et al. 2023). The peaks in region at 478.89 cm^−1^ and 458.33 cm^−1^ attributed to the metallic character of the sample (Yadav et al. 2015). Likewise, IR spectrum (Fig. 5B) of ZnONP illustrated prominent peaks at 3276.04 cm^−1^and 2923.16 cm^−1^ which corresponds to O–H hydroxyl group and side chains of C-H vibrations respectively (Saravanan et al. 2018). Peak at 2162.18 cm^−1^ was resulting from C $\equiv$ C symmetric alkenes (Ramesh et al. 2021). The peak 1724.27 cm^−1^ and 1536.32 cm^−1^ attributes to the stretching vibration due to C = O and presence of aldehydes group (Tyagi et al. 2020). At 1632.74 cm^−1^ and 1015.41 cm^−1^ of absorbance the peak indicates C = O stretching as a result of amine presence and presence of alkyl groups due to C-O stretching vibrations (Zafar and Iqbal 2022). The peak at 1451.59 cm^−1^ suggests the possibility of nitrosamines and vibrations of C-O stretching (Sangeeta et al. 2024a). The peak at 1230.5 cm^−1^ corresponds to C–C bond (Morowvat et al. 2023). The weak peaks 543.65 cm^−1^, 471.93 cm^−1^ and 457.06 cm^−1^ indicated the vibration of bonds between zinc molecule and oxygen (Revankar et al. 2023b). Similarly, the presence of peaks at 2924 cm^−1^ and within the 1350–1750 cm^−1^ range indicates the presence of aliphatic C-H vibrations and secondary protein structures, respectively. These spectral features are characteristic of proteins and their associated functional groups (Shettar et al. 2023a). Another study by (Islam et al. 2023) showed the similar peaks at 1792 cm^−1^ which corresponds to C = O.

#### TGA analysis of L-Glutaminase-capped nanoparticles

The stability, moisture content and composition of synthesized L-Glutaminase capped AgNP and ZnONP was examined using thermogravimetric analysis. Figure 5C illustrates the TGA curve for AgNP (50 mM) heated to 1000 ºC. The curve exhibited five-stage weight loss pattern for AgNP, with the initial weight loss of only 0.83% when temperature was raised to 235.87 ºC indicating good thermal stability. During the second stage and third stage, 3.86% and 3.048% of the weight loss was observed between 280.33 and 325.95 ºC and 325.95 – 691.81 ºC respectively which contributes to the nanoparticle’s dehydration. Initial weight loss of 3.091% during fourth stage when heated between 691.81 and742.5 1 ºC was due to the removal of moisture, hydroxide and ethanol. When heated above 750 ºC, critical weight loss of 28.64% was observed corresponding to the decomposition of the carbonates, sulphates and organic biomolecules (Islam et al. 2023). The TGA curve of ZnONP (Fig. 5D) exhibited three stage weight loss with the initial loss of 10.40% when temperature was raised to 174.50 ºC. Significant weight loss of 47.80% was observed between 275.98 and 370.94 ºC owing to the degradation of organic biomolecules. Following the weight loss of 24.49% was evidenced when temperature was elevated to 1000 ºC. A similar weight loss pattern was previous studied for subtilisin-capped nanoparticles (Shettar et al. 2023a) and synthesized AgNP and ZnONP with keratin hydrolysate (Revankar et al. 2023a, b). In a study (Mahalakshmi 2024), the TGA curve indicated the oxidative characteristic properties of NiO NPs and demonstrated 700 C as the calcination temperature of NiO.

#### SEM–EDS analysis of L-Gutaminase capped nanoparticles

To visualize the surface morphology and the composition of the nanoparticles SEM–EDX was carried out. In Fig. 5E, the shape of the AgNP particles synthesized from L-Glutaminase exhibited an opaque, spherical nature and some multi-shaped and maintained uniform distribution overall. Using EDX mapping, a trace element map was generated with C (27.3%), O (11.3%), Al (1.0%), Cl (28.9%) and Ag (31.4%), as shown in Fig. 5F. Similarly, a study by (Zafar and Iqbal 2022) has represented Ag L (63.84%) and O K (36.16%) having spherical, cubical, bean-like, and irregular shapes. A strong signal of 3 keV was observed, indicating strong signals for Ag atoms, which arises from a phenomenon called surface plasmon resonance and is often used for the absorption of silver nanocrystals and metallic acid (Mechouche et al. 2022). Similarly, highest percentage of 48.7% of Ag was observed by the study conducted by (Fouda et al. 2020) for silver nanoparticles synthesized by endophytic *streptomyces spp*. The SEM images in Fig. 5G of synthesized ZnONP showcased irregular crystalline structure and were dispersed throughout the surface. The EDX spectra revealed oxygen and carbon peaks, which could be attributable to the residual components surrounding the NPs or from the SEM grid employed for sample preparation. Additionally, peaks corresponding to sodium (Na), phosphorus (P), and nitrogen (N) were observed throughout the spectra. A peak obtained at 1 keV confirmed the existence of Zn in the synthesized nanoparticles (Fig. 5H) (Suba et al. 2021).

#### X-ray diffraction analysis of nanoparticles

XRD unveils the crystalline structure of compounds by analyzing the interactions of X-rays with their tiny lattices. L-Glutaminase capped ZnONP were characterized by XRD revealing their crystal structure and evidenced by the presence of Bragg reflections. Comparing with the “Joint Committee on Powder Diffraction Standards” now called “International center for Diffraction Data”, file no 89–7102, the X-Ray diffraction pattern revealed 3 prominent at 31.62 (101), 43.73(102) and 62.75(103), all within the 2θ range of 20 to 70º (Fig. 5I). The presence of these characteristic peaks confirms the hexagonal close-packed (hcp) crystal structure of the sample (Balraj et al. 2017). The medium crystalline size of the L-Glutaminase capped ZnONP was measured using Debye–Scherrer equation. Mathematically, this can be represented as,

$${\text{D}}\, = \,{\text{K}}\lambda /\beta {\text{cos}}\theta$$

where K is the shape factor (0.89),λ is the X-ray wavelength (here λ is 1.54Å), β is the full width at the half maxima (radian), D is the crystalline sizeandand θ is the Bragg angle (2θ) (Shettar et al. 2023a). In a similar study, ZnONP from native *Bacillus cereus* has revealed the crystalline structure and shown similar 2θ values. The X-ray diffraction analysis of biogenic ZnONPs revealed a crystalline structure with characteristic peaks at 32.50° (100), 34.74° (002), 36.69° (101), 47.33° (102), 56.90° (110), 64.32° (112), and 74.90° (202), which correspond to the standard reference pattern for ZnONPs (Ahmed et al. 2021).

#### Atomic force microscopy (AFM) analysis

AFM exceptional resolution and 3D visualization helps to understand the size, shape, and surface topology of molecules. The AgNP were scattered throughout and smooth layers was observed. The higher silver concentration results in the formation of smoother layers. Characterization revealed that the AgNP were within the range of 3–8 nm (Karthik et al. 2014). Figure 5J confirms the formation of AgNP and their tendency to aggregate. Similar studies (Ashraf et al. 2021) revealed that the synthesized AgNP from an extracellular protein of *Enterobacter cloacae* were spherical in shape. Study (Nejad et al. 2024) confirmed the similar results of presence of spherical and triangular AgNPs synthesized from *Streptomyces spp.* isolates KM59, SM88 and KM93 differing in particle sizes. The synthesized ZnONP confirms uneven spherical appendages of different lengths and it ranges from 15–25 nm (Fig. 5K). Another study (Rajeswaran et al. 2023) revealed average particle size of zinc oxide nanoparticles synthesized from *Streptomyces sp. SRT21* as 29.0 ± 1.0 nm with different angles and a peak height. Similar studies (Sangeeta et al. 2024b) validates zinc oxide nanoparticle synthesized from *Talaromyces islandicus* that the average size range falls within the range of 38 to 52 nm.

### Functional characterization of L-Glutaminase

#### Antimicrobial activity of L-Glutaminase and synthesized nanoparticles

The wild L-Glutaminase, AgNP (5 mM, 10 mM and 50 mM) and ZnONP (10 mM and 50 mM) were evaluated for the antibacterial activity against all the six selected pathogens*.* L-Glutaminase capped AgNP (50 mM) from *S. roseolus* exhibited the highest inhibitory activity of 45 $\pm$ 0.5 mm for *B. subtilis* and 33 $\pm$ 0.8 mm for *E. coli,* respectively as indicated in Table 1. Lowest inhibition of 13 $\pm$ 0.1 mm and 17 $\pm$ 0.2 mm for 5 mM and 10 mM of AgNP respectively against *B. subtilis* and 15 $\pm$ 0.1 mm and 18 $\pm$ 0.2 mm for 5 mM and 10 mM of AgNP respectively against *Salmonella typhimurium* was observed*.* L-Glutaminase capped ZnONP (50 mM) exhibited highest inhibition of 30 $\pm$ 0.3 mm for *E. coli* and 25 $\pm$ 0.5 mm for *B. cereus* and lower inhibition of 8 $\pm$ 0.3 mm and 10 $\pm$ 0.1 mm for 10 mM and 50 mM of ZnONP against *S. typhimurium*. The increasing concentration of the nanoparticles exhibited increased inhibitory potential. The wild L-Glutaminase showed highest antibacterial activity of 26 $\pm$ 0.3 mm and lowest of 13 $\pm$ 0.4 mm against *B. subtilis* and *S. aureus,* respectively (Table 2). L-Glutaminase capped nanoparticles showed the highest inhibitory activity compared with the wild L-Glutaminase. The zone of inhibition for cefixime was between 14.0 ± 0.8 mm and 27.0 ± 0.4 mm. In consent with our results, (El-Borai et al. 2023) research revealed the antibacterial activity of purified *Pseudomonas sp.* RAS123 L-Glutaminase with 35.60 $\pm$ 1.80 mm against *B. subtilis* and 26.30 $\pm$ 1.50 mm against *E. coli.* Likewise, (Chakraborty et al. 2022) depicted the antibacterial activity of *Streptomyces sp.* KS46 by 18 $\pm$ 0.65 mm against *E. coli.* Another study (Pillai et al. 2020) states that ZnONP produced by plant extract of *Beta vulgaris* and *Brassica oleracea var. italica* show the antibacterial activity of 10 mm against *E. coli.* (Eltarahony et al. 2021) showed that the AgNP produced by *Streptomyces sp.* possessed antibacterial activity of 8 $\pm$ 0.1 mm against *E. coli.* Similarly, a study (Prema et al. 2017) observed a ZOC of 12.40 $\pm$ 0.83 mm for AgNP against *E. coli* without stabilizer, while 15.30 $\pm$ 0.92 mm with carboxymethyl cellulase as a stabilizer, while study of green synthesis of AgNPs using *Nostoc linckia* exhibited 8.67 mm and 16.67 mm for 1 mM of AgNP against *B. subtilis* and *E.coli* respectively (Vanlalveni et al. 2018). The presence of the AgNP enhances the effectiveness of antibiotics against *B. subtilis* and *E.coli* by eight fold (Prasher et al. 2018).

**Table 2:** Antibacterial activities of L-glutaminase and L-glutaminase capped AgNP and ZnONP

AMA	Samples	Gram Positive			Gram Negative		
		*Bacillus subtilis*	*Staphylococcus aureus*	*Bacillus cereus*	*Escherichia coli*	*Pseudomonas aeruginosa*	*Salmonella typhimurium*
Inhibition zone diameter (mm ± SD)	L-glutaminase	26 ± 0.3	13 ± 0.4	23 ± 0.1	24 ± 0.2	21 ± 0.3	12 ± 0.5
	AgNP (5 mM)	13 ± 0.1	17 ± 0.8	17 ± 0.5	21 ± 0.4	19 ± 0.5	15 ± 0.6
	AgNP (10 mM)	17 ± 0.2	18 ± 0.6	19 ± 0.4	25 ± 0.6	20 ± 0.7	18 ± 0.3
	AgNP (50 mM)	45 ± 0.5	35 ± 0.4	18 ± 0.6	33 ± 0.8	22 ± 0.5	20 ± 0.7
	ZnONP (10 mM)	15 ± 0.9	18 ± 0.8	19 ± 0.7	24 ± 0.5	11 ± 0.7	8 ± 0.3
	ZnONP (50 mM)	20 ± 0.4	23 ± 0.3	25 ± 0.5	30 ± 0.3	15 ± 0.2	10 ± 0.1
	Cefixime	27 ± 0.4	14 ± 0.8	22 ± 0.5	20 ± 0.2	16 ± 0.7	14 ± 0.5

The MIC of L-Glutaminase and L-Glutaminase capped nanoparticles was studied against all the specific six pathogens using the CLSI microbroth dilution method where against *P. aeruginosa* exhibited highest MIC of 0.45 µg/mL suggesting strong antibacterial susceptibility and lowest MIC of 0.09 µg/mL was exhibited against *B. cereus* for L-Glutaminase (Table 3).Several studies explained the mechanism of nanoparticles on the microbial cells, (Konda et al. 2024) studied green synthesis of AgNPs which were treated to *P. aeruginosa* at various inhibitory concentrations to analyse the changes in bacteria’s metabolic profile. It showed prominent alterations in peptidoglycan biosynthesis pathway which may be due to damage to cell well by synthesized AgNPs. There was a depletion of redox factors like NAD(H), NADP(H) and FMN and it also induced global metabolite changes via TCA cycle and glycolysis. (Radzig et al. 2013) reported inhibition of biofilm formation of *Escherichia coli*, where the viability of the cells was reduced by AgNPs stabilized by hydrolysed casein peptides. A study (Mendes et al. 2022) involved exposing *B. subtilis* expressing FtsZ-GFP, an ancestral tubulin which only exerts its function dependent on GTP to ZnONPs. In comparison with the control group, where the cells exhibited intact bars perpendicular to the long axis of rods, the treated cells displayed no disruption of the divisional ring. In case of *E. coli,* the cytoplasmic membrane was disrupted when exposed to fluorescence microscopy. (Xiong et al. 2022) showed elevated antibacterial effect of enzyme-Ag-polymer nanocomposites that constituted of lower enzyme and silver dosages and also suggested a synergistic effect of polymer, enzyme and silver through a tightly encapsulated invasion bactericidal mechanism.

**Table 3:** Minimum inhibitory concentration (MIC) of L-glutaminase and L-glutaminase capped AgNP and ZnONP

MIC	Samples	Gram Positive			Gram Negative		
		*Bacillus subtilis*	*Staphylococcus aureus*	*Bacillus cereus*	*Escherichia coli*	*Pseudomonas aeruginosa*	*Salmonella typhimurium*
MIC (µg/mL)	L-glutaminase	0.09	0.9	0.22	0.22	0.45	1.8
MIC (Mm)	ZnONP (10 mM)	0.06	0.09	0.6	0.090.22	0.9	1.8
	AgNP (5 mM)	0.6	0.45	0.45	0.45	1.8	4.5

#### Antioxidant potential

**Fig. 6:**
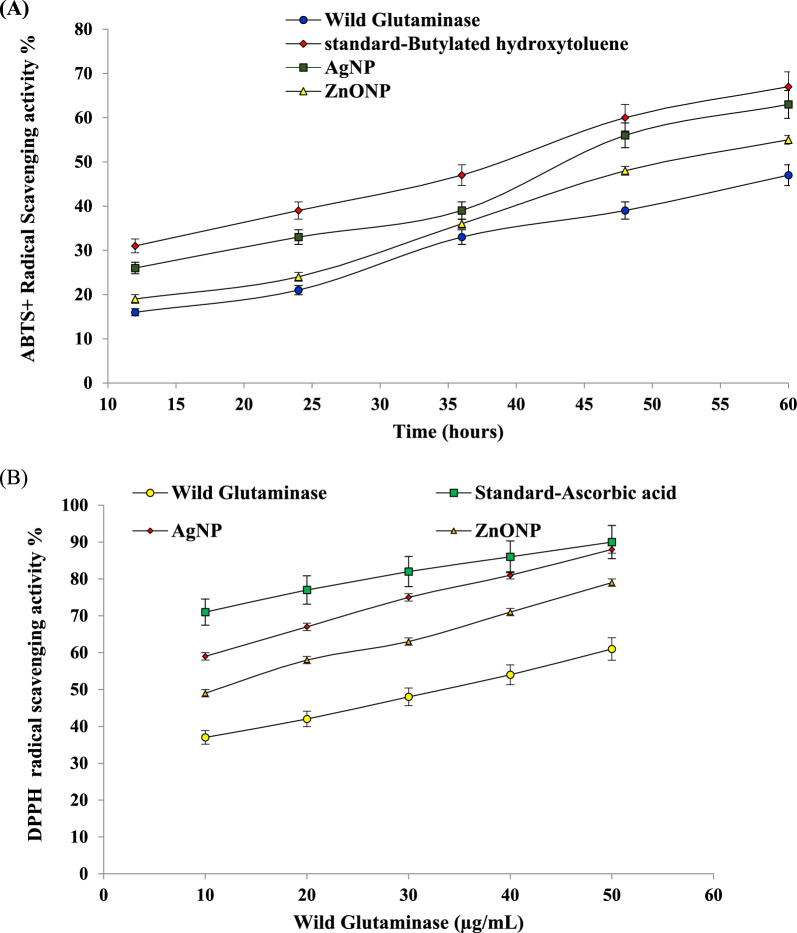
Antioxidant activities of purified recombinant L-Glutaminase and L-Glutaminase capped nanoparticles by **A** ABTS assay and **B** DPPH radical scavenging activity

The indication of potential antioxidant properties was assessed using in-vitro antioxidant assays ABTS and DPPH as they offer a simple, quick assessment of free radical scavenging ability of the purified L-Glutaminase, AgNP and ZnONP. The results of the ABTS assay revealed a progressive increase with respect to time. The inhibition percentage of 16–63% was observed during the period of 12–60 h period. The highest inhibition of 63%, 55% and 47% was recorded for AgNP, ZnONP and L-Glutaminase, respectively, whereas the standard BHT showed the highest inhibition percentage of 67%, as shown in Fig. 6A. In comparison with the L-Glutaminase of 47% inhibition, nanoparticles exhibited stronger antioxidant activity of 63% and 55% for AgNP and ZnONP, respectively. The antioxidant activity was also analyzed by performing DPPH assay with range of concentrations (10–50 µg/mL) of AgNP, ZnONP and purified L-Glutaminase. Inhibition of 88%, 79% and 61% for AgNP, ZnONP and L-Glutaminase was recorded at 50 µg/mL as compared to 90% with ascorbic acid (standard) with IC_50_ value of 8.47 µg/mL, 17.24 µg/mL and 31.25 µg/mL respectively. A positive correlation between concentration and percent radical scavenging activity was observed, indicating a dose-dependent response as illustrated in Fig. 6B. Previous studies by (Unissa et al. 2018) L-Glutaminase isolated from *A. flavus* KUGF009 demonstrated free radical scavenging activity using the ABTS method. The IC_50_ value for L-Glutaminase (98.53 µg/mL) was higher compared to the standard L-ascorbic acid (IC_50_ = 80.52 µg/mL), suggesting a weaker scavenging activity of L-Glutaminase. Another study (Adebayo et al. 2019) also demonstrated concentrated dependent ABTS scavenging activity in plant mediated Ag-NPs ranging from 54.43 to 85.15%. Another study (Ihsan et al. 2023) examined the ABTS radical scavenging activity of nanoparticles derived from *Curcuma zedoaria* and *Momordica charantia*. They observed the highest activities of 42.79% and 46.05% for *M. charantia* and *C. Zedoaria* nanoparticles at 1000 µg/mL, respectively. In line with the results obtained from DPPH analysis, studies have shown that L-Glutaminase from *A. versicolor* exhibited dosage-dependent antioxidant activity (Awad et al. 2021) with 50 µg/mL of IC_50_ value compared to 31.5 µg/mL of L-ascorbic acid. In another study, the halophilic bacteria isolate AD23 has high free radical scavenging activity of 5.48 µg/mL IC_50_ value in comparison with the standard of 6.35 µg/mL (Rathakrishnan and Gopalan, 2022). Similarly, (El-Sousyet al. 2021) reported 0.8 mg/mL of IC_50_ value for crude L- Glutaminase from *Bacillus subtilis* NRRL 1315 compared to 0.65 mg/mL of the standard. Similar study (Safawo et al. 2018) examined the antioxidant activity of ZnONP which revealed IC_50_ of 125 µg/mL. Another study examined AgNPs secreted from the extract of *A. millefolium* showed the highest potential of scavenging DPPH radicals with IC_50_ of 7.03 µg/mL as compared with standard L-ascorbic acid of 4.29 µg/mL (Yousaf et al. 2020). In a study (Lyu et al. 2014), the proteins implanted in metal–organic frameworks through coprecipitation method showed high peroxidase activity where, Cyt*c* was embedded in ZIF-8 suggesting its applications biosensors.The antioxidant properties of nanoparticles emerge due to the interactions that occur among the antioxidant molecules derived from biological entities and the surface area of synthesized nanoparticles that execute their action synergistically. For example, the antioxidant attributes of AuNPs was reported to undergo the preventive mechanism through catalase mimic behavior and superoxide mimic behaviors and the chain-breaking mechanism through transfer of electrons and protons to alkylperoxyl radicals leading to the formation of ROOH (Suliasih et al. 2024).

#### In-vitro cytotoxicity assessment

**Fig. 7:**
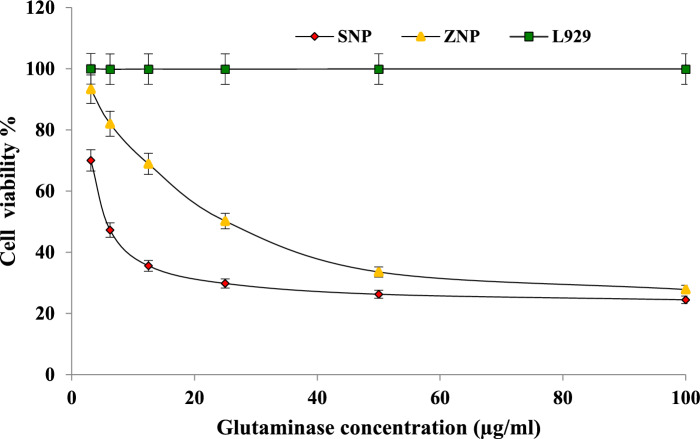
Cytotoxicity activity of purified recombinant L-Glutaminase capped nanoparticles against L929 cell line and breast cancer cell line MCF-7

The pronounced side effects of chemotherapy in cancer treatment calls for the need of alternative treatments such as, employment of enzymes and enzyme capped nanoparticles-based treatment approaches. The cytotoxic effects of purified L-Glutaminase, L-Glutaminase capped ZnONPsand AgNPs were evaluated against human breast cancer cells. A range of concentrations, from 6.25 to 200 µg/mL, was used to assess their potential toxicity. Doxorubicin, a well-established anticancer drug, served as the positive control for comparison. The cytotoxic effect of L-Glutaminase and capped NPs is evidenced in Fig. 7 where a dose-dependent effect is prominently observed for all the treatments. The % cell viability reduced as the concentration of the enzyme and NPs elevated. Comparatively the L-Glutaminase capped AgNP showed a pronounced higher effect with IC_50_ value of 8.13 µg/mL. The IC_50_ value for L-Glutaminase and L-Glutaminase capped ZnONP was found to be 57.17 µg/mL and 28.31 µg/mL respectively. IC_50_ value is an indicative of effective concentration of anticancer agent required to induce 50% cell inhibition. The inhibition rates were more pronouncing when the L-Glutaminase concentration was greater than 50 µg/mL. L-Glutaminase capped nanoparticles exhibited a concentration-dependent cytotoxic effect, with a drastic decline in cell viability observed at concentrations exceeding 20 µg/mL. This suggests a more pronounced impact on cell viability at higher concentrations. L-Glutaminase did not reflect any measurable effect on tested normal cells (L929) which is an indicative of its non-effectiveness against normal cells. An IC_50_ value of 3.6 µg/mL was observed for the standard (Doxorubicin). The outcomes imply promising influences of L-Glutaminase and its NPs on the proliferation of breast cancer cells suggesting its promising attributes as anticancer representative in enzyme therapy. Glutamine plays a significant role in cellular metabolic activities by fueling the required nitrogen for various biosynthetic pathways of nucleotides and amino acids. Owing to the malfunctioning of metabolism in cancer cells exerts high demand for glutamine. The action of L-Glutaminase triggers the hydrolytic response by producing ammonia and glutamic acid thereby starving the cancer cells for glutamine. This effective mechanism makes L-Glutaminase a crucial therapeutic agent against different cancers. Recent studies report the anticancer potential of L-Glutaminase derived from *Pseudomonas sp* RAS123, demonstrating activity against MCF-7 cell line with 195 µg/mL of IC_50_ value (El-Borai et al. 2023). L-glutaminase from *Bacillus sp*. DV2-37 (Gomaa 2022a, b) and *Streptomyces rochei* SAH2_CWMSG (Awad et al. 2019) revealed IC_50_ value of 3.5 µg/mL and 405.1 µg/mL respectively against MCF-7. Study reported reasonable anticancer activity of silver nanoparticles with IC_50_ value of 138.30 µg/mL synthesized using *Streptomyces* sp. PG12 (Pallavi et al. 2022). The silver nanoparticles from plant extract *Heliotropium bacciferum* showed IC_50_ value of 5.44 µg/mL for MCF-7 (Khan et al. 2021). The synthesized Ag-NPs from *Streptomyces enissocaesilis* BS1 (Shaaban et al. 2024) and *Streptomyces rochei* HMM13 (Abd-Elnaby et al. 2016) indicated promising antiproliferative activities. Where in the case of zinc oxide nanoparticles synthesized using *Streptomyces* sp. exhibited inhibition with 15.6 µg/mL IC_50_ value (Balraj et al. 2017). While zinc oxide nanoparticles exhibited promising antiproliferative activity, copper oxide nanoparticles synthesized using *Macroptilium lathyroides* leaf extract displayed a comparable effect with 71.02 µg/mL of IC_50_ value (Prabu and Losetty, 2024). Apoptosis seems to be an important aspect of normal cell development and is frequently dysregulated in cancer. Compounds from biological sources have potential features to induce apoptosis and inhibit the cell cycle of MCF-7 in phase G_2_-M thereby restricting the growth of cancer cells as reported (Hasibuan et al. 2024) with ethyl acetate fraction of *Vernonia amygdalina* Delile to be prospective anticancer agent against the MCF-7/HER-2 cell line.The current result reflects the ability of L-Glutaminase in enzyme therapy-based treatment of cancers.However, future studies are required to evaluate the *in-vivo* therapeutic effects of L-Glutaminase in animal models. Studies on pharmacokinetic and pharmacodynamics properties are essential for extended biomedical applications.

## Conclusion

L-Glutaminase was produced from *Streptomyces roseolus* strain ZKB1 and subsequent immobilized on agar and agarose matrices that revealed enhanced recycling stability. The immobilized enzyme was employed for the synthesis of L-Glutaminase-capped silver and zinc oxide nanoparticles. Comprehensive characterization of these nanoparticles, including FTIR, TGA, XRD, SEM–EDS, and AFM investigation, revealed information about their physiochemical properties. The resultant nanoparticles also exhibited promising biological activities, such as antibacterial, antioxidant, and cytotoxic properties. These findings highlight the potential applications for these nanoparticles in variety of disciplines.

## Data Availability

All data generated and analyzed during the current study are included in the article.

## Abbreviations

NCIM: National collection of industrial microorganisms
MTCC: Microbial Type Culture Collection and Gene Bank
PCR: Polymerase chain reaction
EF: Efficiency of immobilization
*V*_*max*_: Maximum velocity
*K*_*m*_: Michaelis–Menten constant
*k*_*cat*_: Turnover number
*k*_*cat*_*/K*: Catalytic efficiency
AgNP: Silver nanoparticles
ZnONP: Zinc oxide nanoparticles
FTIR: Fourier transform infrared spectroscopy
KBr: Potassium bromide
TGA: Thermogravimetric analysis
SEM- EDX: Scanning electron microscope with energy-dispersive X-rays
XRD: X-ray diffraction
AFM: Atomic force microscopy
CLSI: Clinical and Laboratory Standards Institute
MIC: Minimum inhibitory concentration
ABTS: 2,2–Azinobis– (3–Ethylenebenzothiozoline–6–Sulfonic Acid)
BHT: Butylated hydroxytoluene
DPPH α: α-Diphenyl-β-picryl-hydroxyl
MTT: (3-[4,5-Dimethylthiazol-2-yl]-2,5 diphenyltetrazolium bromide)
PBS: Phosphate buffer saline
SPR: Surface plasmon resonance
